# Hybrids versus parental species: insights from wing phenotype similarities and differences in triatomine insects

**DOI:** 10.3389/finsc.2025.1547963

**Published:** 2025-03-11

**Authors:** Álvaro Lara, María Laura Hernández, César A. Yumiseva, Mario J. Grijalva, Anita G. Villacís

**Affiliations:** ^1^ Centro de Investigación para la Salud en América Latina (CISeAL), Facultad de Ciencias Exactas y Naturales, Pontificia Universidad Católica de Ecuador, Quito, Ecuador; ^2^ Unidad Operativa de Vectores y Ambiente (UnOVE), Centro Nacional de Diagnóstico e Investigación en Endemo-Epidemias, Administración Nacional de Laboratorios e Institutos de Salud Dr. Carlos Malbrán (CeNDIE-ANLIS Malbrán), Santa María de Punilla, Córdoba, Argentina; ^3^ Consejo Nacional de Investigaciones Científicas y Técnicas (CONICET), Cordoba, Argentina; ^4^ Infectious and Tropical Disease Institute, Department of Biomedical Sciences, Heritage College of Osteopathic Medicine, Ohio University, Athens, OH, United States

**Keywords:** Ecuador, Chagas disease, hybrids, morphometrics, triatomines

## Abstract

**Introduction:**

The genus *Panstrongylus* is one of the most important within the subfamily Triatominae, which includes vectors of *Trypanosoma cruzi*, the etiological agent of Chagas disease (CD). In particular, *Panstrongylus chinai* and *P. howardi* have drawn attention for their role in disease transmission. These species exhibit notable ecological and morphological differences. Previous studies have investigated aspects such as morphometry, cytogenetics, and ecological niches, including experimental crosses between these species that resulted in viable F1 hybrids. However, no F2 generation was produced, as the eggs laid were empty and failed to hatch, limiting the study to F1 hybrids.

**Methods:**

We analyzed wing morphometric traits (size and shape) from 262 individuals, including *P. chinai*, *P. howardi*, and their hybrids, using geometric morphometry techniques. This study aimed to build upon previous findings by analyzing the wing morphometric and environmental adaptations of *P. chinai*, *P. howardi*, and their hybrids (♀*P. howardi* × ♂*P. chinai*) to determine whether the hybrids exhibited similarities in wing size and shape, regardless of maternal or paternal phenotype.

**Results:**

Differences in centroid size were observed between the parental species, with *P. howardi* having a larger size, but no significant differences were found among the hybrids. Females showed greater shape similarity between *P. howardi* and the ♀*P. chinai* × ♂*P. howardi* hybrids, while males showed similarity among hybrids. Discriminant analysis was more effective for distinguishing parental groups than with hybrids. The *K*-means algorithm successfully classified the parental species and hybrid groups, although with low assignment percentages and a different number of groups than expected.

**Discussion:**

The smaller wing size in hybrid offspring may indicate lower fitness, potentially due to genetic effects or reduced viability. Geometric morphometry effectively distinguishes parental species from hybrids, supporting previous research in Triatominae. The study suggests that environmental and reproductive pressures may influence these species and explores the dispersive capabilities of triatomines, contributing to the understanding of hybridization processes.

## Introduction

Chagas disease is a neglected tropical disease caused by the protozoan *Trypanosoma cruzi* and is transmitted mainly through the feces of triatomine bugs. It affects six to seven million people (1) and poses significant health challenges, particularly in Central and South America (2). In Ecuador, approximately 230,000 people are infected, and 6.2 million people are at risk of infection (3).

The subfamily Triatominae comprises more than 155 species (4–9). In Ecuador, 16 species have been reported across 18 of the 24 provinces (10). The main vectors in the country are *Rhodnius ecuadoriensis* Lent & Leon, 1958, and *Triatoma dimidiata* (Latreille, 1811) though most studies have focused on the former rather than the latter (11–16). However, the epidemiological importance of several species considered secondary vectors has increased due to their fast expansion into human-inhabited areas (17) and changing climate conditions (18, 19). The *Panstrongylus* genus is becoming more significant in Chagas disease (CD) transmission. Key characteristics of some species within this genus include: (i) a short to medium life cycle (149.5 to 531 days), (ii) prolonged contact with the host and a quick response to their presence, (iii) the capacity to consume large volumes of blood, (iv) frequent defecation while feeding, and (v) high rates of *T. cruzi* infection (20). Six species of this genus have also been reported in Ecuador: *Panstrongylus chinai* (Del Ponte, 1929), *P. howardi* (Neiva, 1911), *P. geniculatus* (Latreille, 1811), *P. lignarius/P. herreri* (Walker, 1873), and *P. rufotuberculatus* (Champion, 1899) (10, 14, 21).

*Panstrongylus chinai* is primarily found in domiciliary habitats (22) in Loja and El Oro provinces (16, 20, 23), while *P. howardi* is endemic to the province of Manabí, typically inhabiting peridomiciliary habitats (21, 24) and occasionally occurring in sylvatic environments (25). The importance of these species as vectors is highlighted by their increasing spread and adaptation to human-inhabited areas.

Regarding its morphology, *P. chinai* can be completely black and/or brown. Its scutellar process is elongated, distally sharpened, and subcylindrical. The body length ranges from 22 to 27 mm, the pronotum width from 5.5 to 7 mm, and the abdomen can be up to 12 mm wide (26). In contrast, *P. howardi* displays a brown-orangish coloration, has a second rostral segment longer than the first, and lacks denticles on its femur. Its body length ranges from 25 to 29 mm, the pronotum width from 6 to 6.5 mm, and the abdomen width from 8.5 to 11 mm (26). Despite their differences, these species are closely related, and hybridization has been observed in experimental crosses (14). To investigate this, multiple methods were employed, including antennal morphology, geometric morphometrics of the head, wings, and eggs, cytogenetic analysis, ecological niche modeling, and experimental crosses. The crosses produced viable F1 hybrids with a *P. howardi*-like coloration but a *P. chinai*-like body size. However, further crosses between F1 hybrids resulted in no copulation or viable offspring. Hybridization, which generates genetic variation and novel traits, may underlie these findings. The sterility or nonviability of F1 hybrids could result from genetic incompatibilities, loss of local adaptations, or disruption of co-adapted genes (27).

Geometric morphometrics is a method that quantifies morphological variation and was crucial in this study, allowing for the analysis of traits influenced by both genetic and environmental factors (28, 29). In insect studies, it is important to recognize that adult morphological markers are fixed, reflecting both life history and environmental adaptations. The environment in which an insect develops can lead to variations in its phenotype, behavior, physiology, and morphology, affecting its dispersal potential and influencing factors such as habitat selection, host preference, and vector capacity (30, 31). In triatomines, geometric morphometrics has been applied to various taxonomic issues, including habitat markers, population structure, domiciliation, flight characteristics, and the effects of insecticide exposure and resistance (12, 14, 32–39). It has also been particularly useful in studying hybrids to explore speciation hypotheses, identify closely related species, and analyze morphological traits and morphometric changes associated with dispersal (9, 40–42).

Geometric morphometrics is a powerful tool for studying the phenotypic traits of triatomines, including wing morphology. This method allows for the quantification of morphological variation and provides insights into ecological adaptations, dispersal potential, and vector capacity. Furthermore, it has been vital in differentiating medically important species in Ecuador, such as *R. ecuadoriensis* (12), *P. rufotuberculatus* (43), *P. chinai*, and *T. carrioni* (Larrousse 1926) (44). This study aims to characterize the populations of *P. chinai*, *P. howardi*, and their experimental hybrids by comparing wing morphometric traits and exploring their potential environmental adaptations. The hybrids, which exhibit *P. howardi*-like coloration and *P. chinai*-like body size, are expected to have wing traits similar to *P. howardi* due to their dominant phenotype, along with intermediate wing characteristics between the two parental species. Additionally, hybrids may display phenotypic traits aligning with one of the parental species. This research highlights the morphological variability in hybrids and examines how phenotypic inheritance and sexual variation may influence their characteristics.

## Methodology

### Collection of individuals and description of the study area

The *Panstrongylus chinai* and *P. howardi* individuals (both parental) used in this study were collected from two regions of Ecuador—the Southern Andean region and the Central Coastal region—specifically from five rural communities in Loja province (Bellamaria [−4.194885, −79.610214, and 1,106 m above the sea level {masl}]; Guara [− 4.25007, − 79.57979, and 1,081 masl]; Huayco [−4.091017, −79.322817, and 1449 masl]; Vega del Carmen [−4.11105, −79.7074, and 1,612 masl]; and Tacoranga [−4.106107, −79.590123, and 1,141 masl]) and one rural community of the Manabí province (Bejuco [−0.949592, −80.3365, and 383 masl]). These individuals were collected under permits No. 002-07-IC-FAU-DNBAPVS/MA, No. 006-RM-DPM-MA, No. 006-IC-FLO-DPL-MA, No. 008-RM-DPM-MA, No. 008-IC-INSEC-DPL-MA, No. 010-IC-FAU-DNBAPVS/MA, and No. 016-07 IC-FAU-DNBAPVS/MA, all granted by the Ministry of Environment of Ecuador.

Loja province features a rugged, hilly landscape with altitudes ranging from 700 to 3,700 m. Average temperatures range from 15.4°C to 26.6°C, with annual rainfall varying from 0.2 to 200 mm (45). The province plays a significant economic role, with 61% of its land dedicated to agriculture and livestock, producing crops such as corn, sugarcane, coffee, and rice (46). Traditional homes are constructed with adobe walls, dirt floors, and ceramic tile roofs to help regulate temperature (47).

Manabí province, characterized by flatter terrain and a 350-km coastal expanse, has elevations reaching up to 500 m. Its average temperature ranges from 20.3°C to 26.6°C, with rainfall varying from 10 to 5,000 mm (45). The province plays a key role in Ecuador’s economy, with 84% of its land used for sugarcane, plantain, and oil palm cultivation (46). Traditional homes are constructed using bamboo (caña guadúa) for walls and floors, with roofs made of palm fronds or, occasionally, zinc (24).

While both regions’ housing styles are adapted to local climates and lifestyles, they do not meet health or technical standards, increasing the risk of triatomine infestation and *T. cruzi* infection (47).

### Hybrid crosses and selection of individuals

The collected specimens were transported under mobilization permit number MAE-DNB-CM2015-0030 to the Insectary of the Center for Research for Health in Latin America (CISeAL). The identification of developmental stages and the sex of adult triatomines was performed using a dichotomous key by Lent and Wygodzinsky (4).

These individuals were maintained in an incubator equipped with a dual-chamber system, replicating the original microhabitat temperature and humidity conditions of each province (Manabí: 27°C ± 5°C, 75% ± 5% RH; Loja: 24°C ± 6°C, 70% ± 5% RH) with a 12-h photoperiod in both chambers, as described in Villacís et al. (11) and Santillán-Guayasamín et al. (48) at the Insectary of the CISeAL. For the experimental crosses and the production of hybrids, individuals from the Manabí and Loja communities were used, as described in Villacís et al. (14). Fifth-instar nymphs (NV) of *P. chinai* and *P. howardi* were separated to obtain virgin females for crossing. After emerging as adults, males and females were placed in plastic vials with filter paper to facilitate movement and humidity control. Biweekly blood meals were provided using immobilized pigeons, following protocol 15-H-034, approved by the American Association for Laboratory Animal Science - IACUC. Nine interspecific crosses were performed: five with one female of *P. chinai* and two males of *P. howardi* (♀*Pc* × ♂*Ph*) and four with one female of *P. howardi* and two males of *P. chinai* (♀*Ph* × ♂*Pc*). These pairings were based on the availability of insects to ensure copulation. After F1 adults emerged, nine couples were formed to attempt F2 offspring. However, no copulation occurred between F1 hybrids, and the eggs laid were empty and did not hatch, so only F1 hybrids were used (14).

For morphometric analysis, we extracted the wings of 262 individuals as follows: *P. chinai* (38♀ and 53♂), *P. howardi* (36♀ and 47♂), and 88 hybrids between these two species. All analyses were conducted using only the right wing of male and female individuals from each group.

### Linear measurement of body length of parental species and hybrids

Body size measurements (from the clypeus to the genitalia) were taken for 30 adult individuals from each group using a digital caliper (Didimatic Caliper, Model CD-6”C, Mitutoyo Corporation, Kawasaki, Japan): *Panstrongylus chinai* (15♀ and 15♂), *P. howardi* (15♀ and 15♂), and hybrids (15♀ and 15♂). Each measurement was conducted once, with no replicates.

### Preparation of wings and image digitization

**Figure 1:**
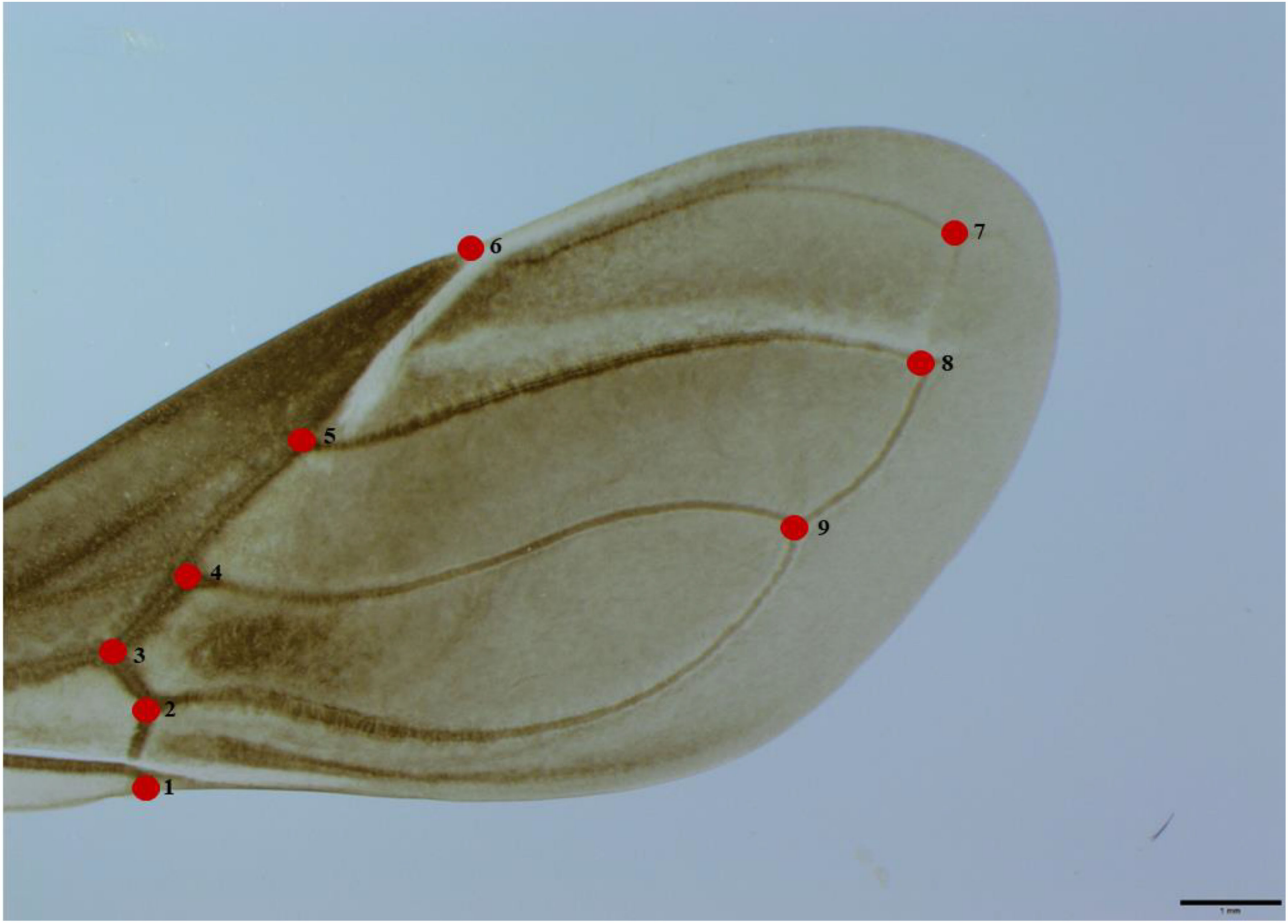
Dorsal view of the right wing of a female *P. chinai*, with nine digitized landmarks.

Each wing was extracted, placed on a slide, and covered with a drop of Hoyer’s medium. A cover slip was added to evenly distribute the medium, and the slide was left to dry for a week until it solidified. Hoyer’s medium was chosen for its ability to clean the tissue, enhance visibility due to its high refractive index, and preserve the structure for up to 20 years (49). Afterward, a digital image of each wing was captured using an OLYMPUS SZX7 stereoscope and a camera (OLYMPUS^®^ CMOS 10.6 MP Microscope Digital Camera, Model SC100, Olympus Corporation, Tokyo, Japan). Nine type I *landmarks* were used for morphometric analyses (**Figure 1**). Given that some wings from the three groups, particularly those from *P. howardi*, were highly transparent, Adobe Lightroom (Adobe Photoshop Lightroom Classic CC v.7.5, Adobe Inc, San Jose, CA, USA) was used to adjust the lighting and enhance the visibility of the landmark locations.

### Repeatability

Before marking landmarks and conducting morphometric analyses, repeatability was tested to assess the accuracy of landmark digitization. Fifteen wing images from *P. howardi*, *P. chinai*, and hybrids of both sexes were randomly selected and digitized twice by the same user. The repeatability index was calculated using Procrustes analysis of variance (ANOVA) to evaluate measurement error. The statistical method used by the XY Online Morphometrics (XYOM) software for shape repeatability estimation followed a model II one-way ANOVA for repeated measurements (50) and Procrustes ANOVA (51).

### Statistical analysis

To obtain shape variables and centroid sizes, generalized Procrustes analyses were performed for each sex using ANOVA (52). Both size and shape variables enabled the subsequent analyses detailed below.

### Size comparisons

The global size of the wings was computed as centroid size (CS), defined as the square root of the sum of the squared distances between the center of the landmark configuration and each individual landmark (53). Wing sizes (CS) were compared between groups (*P. chinai* and *P. howardi* and hybrids ♀*Pc* × ♂*Ph* and ♀*Ph* × ♂*Pc*) for each sex using a one-way ANOVA.

### Shape comparisons

The comparison of shapes between hybrids and parental specimens for each sex was conducted using principal component analysis (PCA) of the Procrustes residuals and discriminant function analysis (DFA). The statistical significance of the analyses on wing size and shape was assessed using a nonparametric permutation test (5,000 runs).

The classification, based on the cross-check test using Mahalanobis Distances, indicates the percentage of individuals correctly assigned to each group (reclassification) (54).

### Characterization

The metric disparity (MD) index quantifies the variance in wing shape. This index provides a single value that measures the variability in a sample’s shape and is calculated based on the Euclidean distances of each configuration relative to the consensus (55). The significance of potential differences in metric disparity between groups was assessed using bootstrap-based statistical methods and the *F*-test for equality of variances.

### Allometry

Size and shape variables were analyzed separately, and their possible relationship, or allometry, was measured using linear regression techniques. Centroid size was regressed against the first two principal components of shape (PC1, PC2) using the XYOM software, which is freely available online (http://xyom.io 54).

### Classification

We use unsupervised analysis techniques to evaluate the assignment of hybrid individuals concerning their parental species. The classification was performed using covariance-based PCA and the *K*-means classification algorithm. PCA is a tool that enhances the visualizations of multidimensional data by removing correlations between variables, which is a common feature of morphometric traits. By creating new uncorrelated variables, PCA graphically illustrates the primary trends structuring the data. The *K*-means algorithm, a blind classification technique, identifies potential natural groupings within the data without relying on predefined labels (56). The analysis primarily relied on inter-individual distances, specifically the Euclidean distance calculated from the raw variables. The predefined number of groups (*K*) was always set to four, corresponding to the two parental species and the two hybrid groups, each derived from a mother of a different species. The group composition determined by the *K*-means algorithm was compared with the pre-established classification, enabling the calculation of the percentage of correct classification. The algorithm was initiated using the “naive sharding centroid” method, and the optimal number of clusters was determined using the “elbow graph” method, which analyzes the average distance to the centroid as a function of *K*. For each sex, the *K*-means algorithm was applied to both shape and size variables.

### Morphometric software

The collection of landmarks and statistical analyses was conducted using several modules of the XYOM platform, a free online program that enables users to follow the entire geometric morphometrics analysis workflow with ease (http://xyom.io 54). It is user-friendly and does not require installation, configuration, or manual updates. Since it is a cloud-based platform, a Google Drive account is necessary to store all the information related to the landmark digitization process. XYOM also uses Plotly to enhance the editing and visualization of its various statistical outputs. Users can download the results and combine multiple output files to create new datasets for further analysis. This feature is particularly useful when working with large datasets (54).

## Results and discussion

Morphometry measures and describes the body morphology of organisms, providing quantitative data on their environmental interactions. Recent advancements have made morphometric analyses more visual and less labor-intensive. Chagas disease vectors, or “kissing bugs”, belonging to the Triatominae subfamily, are widely studied for their morphological plasticity. Research has focused on aspects such as domesticity, food preferences, dispersal, insecticide resistance, speciation processes, and taxonomy (40, 57). In addition, morphological stasis in Triatoma brasiliensis brasiliensis, as observed through geometric morphometrics by Paschoaletto et al. (58), highlights its adaptability to climate changes and global invasiveness. These findings offer valuable insights into the evolutionary patterns of wing morphology in Triatominae as disease vectors.

Geometric morphometrics, based on the quantitative analysis of triatomine wing venation characters, has proven to be a reliable tool for identifying triatomines, particularly when distinguishing morphologically similar species (sibling species or hybrids). Our results support the findings of Villacís et al. (14) and Barnabé et al. (59), confirming that *P. chinai* and *P. howardi* should not be synonymized and that these taxa represent two valid yet closely related species.

This is the first study on wing geometric morphometrics in hybrid individuals of the genus *Panstrongylus*. Hybridization events are usually considered an evolutionary dead end because they frequently result in nonviable or infertile offspring (60, 61). Nevertheless, an alternative approach suggests that natural hybridization generates novel combinations of genes and alleles, fostering favorable genetic conditions for rapid and significant evolutionary changes (40, 62).

Our findings indicate that the hybrids exhibit distinct morphometric characteristics in their wings compared to their parental species. Additionally, the level of phenotypic similarity between the hybrids and their parental species—*P. chinai* or *P. howardi*—depends on the sex combination of the crossings.

### The total body length of the parental species and hybrid

**Figure 2:**
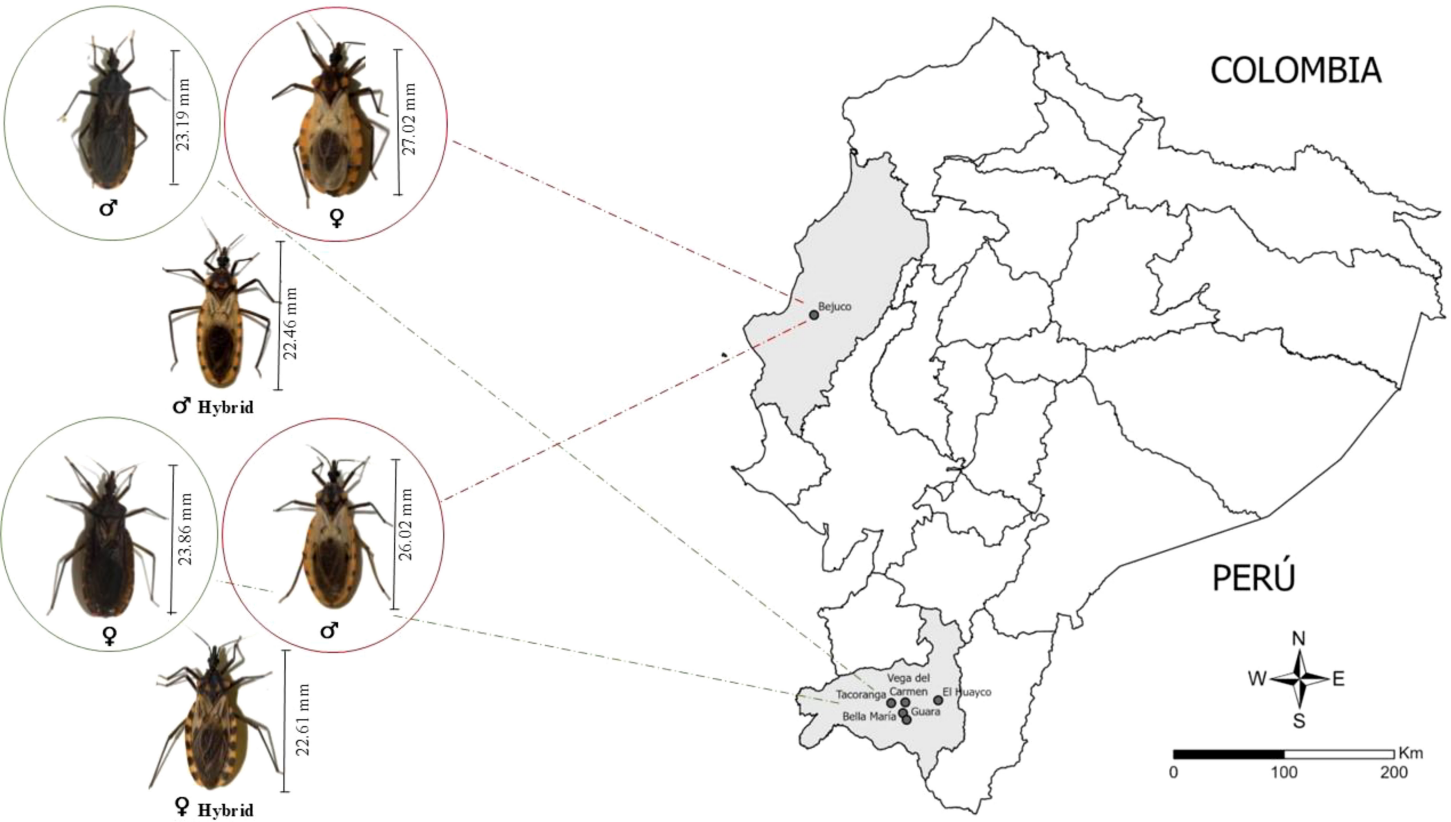
The map of Ecuador shows Manabí and Loja provinces in light gray, with dark grey points indicating the communities where the parental species (*Panstrongylus chinai* and *P. howardi*) were collected. In addition, the figure presents body length measurements of the parental species (*Panstrongylus chinai* and *P. howardi*) and their hybrids.

The body size of the hybrids, whether produced from ♀*P. chinai* × ♂*P. howardi* (♀*Pc* × ♂*Ph*) or ♀*P. howardi* × ♂*P. chinai* (♀*Ph* × ♂*Pc*), was consistently smaller than that of the parental species, *P. chinai* and *P. howardi*. Specifically, the body length of *P. chinai* males and females averaged 23.19 mm ± 0.91 mm and 23.86 mm ± 1.48 mm, respectively, whereas *P. howardi* had larger individuals, with averages of 26.02 mm ± 1.28 mm in males and 27.02 mm ± 1.66 mm in females. The hybrids, on the other hand, showed smaller sizes (♂*Pc* × ♂*Ph*: 22.75 mm ± 1.1 mm in males and 23.41 mm ± 0.8 mm in females; ♀*Ph* × ♂*Pc*: 22.46 mm ± 0.27 mm in males and 22.61 mm ± 0.5 mm in females). However, no significant size differences were detected between the two hybrid groups (**Figure 2**). This smaller body size in hybrids may suggest a potential decrease in overall fitness, aligning with previous findings in other species where hybrid offspring tend to exhibit reduced sizes. According to Dujardin et al. (63), laboratory-reared triatomines often experience a significant reduction in size over successive generations, likely due to a higher survival rate of smaller individuals under such conditions. The inclusion of additional generations, influenced by environmental changes (such as laboratory conditions), may impose a cost on body size (64). Notably, body size is not always related to the centroid size of the structure under study (65).

### How to interpret the size of the hybrids?

**Figure 3:**
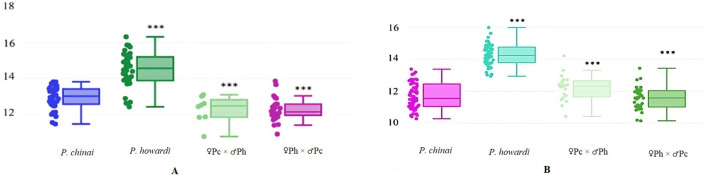
The figure displays the centroid size of the wings of *P. chinai*, *P. howardi*, and their hybrids. The line inside each box represents the median, while the bars indicate the maximum and minimum values. **(A)** Female hybrid from the ♀*Ph* × ♂*Pc*. **(B)** Male *P. chinai* showing the lower fence. In both females and males, asterisks denote significant differences between *P. howardi* and *P. chinai*, as well as between *P. howardi* and hybrids (♀*Pc* × ♂*Ph* and ♀*Ph* × ♂*Pc* crosses). ^***^ *p <* 0.001. IQR, interquartile range.

Fifteen images (right wings of *P. howardi*, *P. chinai*, and hybrid individuals of both sexes) were randomly chosen and digitized twice by the same user. The quality of landmark digitization in our sets showed a repeatability score of 98% for centroid size and 81.9% for shape. Wing size, measured by centroid size, showed notable differences between the parental species, with *P. chinai* having significantly smaller wings (*p <* 0.001) (12.93 mm ± 0.62 mm in females and 11.70 mm ± 0.81 mm in males) compared to *P. howardi* (14.49 mm ± 0.95 mm in females and 14.27 mm ± 0.69 mm in males). However, no significant differences were found in centroid size between the two hybrid groups. Both hybrid groups differed significantly from *P. howardi*, while only the ♀*Pc* × ♂*Ph* hybrid females differed from *P. chinai* (*p* < 0.01) (**Figures 3A, B**). This indicates that size differences in wings between the parental species do not extend to the hybrids, which do not show heterosis in this trait. Hybrid phenotypic traits are typically expected to be intermediate between those of their parental species. For example, hybrids of *Triatoma sherlocki* Papa, Jurberg, Carcavallo, Cerqueira, Barata, 2002, and *T. juazeirensis* Costa & Felix, 2007, showed intermediate sizes, which may enhance their fitness, particularly for dispersal (41). However, in our study, the hybrids exhibited smaller wing sizes, possibly due to lower fitness or dominant genetic effects. Similarly, hybrids between *Drosophila buzzatii* and *D. koepferae* displayed a range of sizes—larger, smaller, and intermediate—indicating that genetic factors such as dominance (66) and epistasis can influence size variability in hybrids (67). Since centroid size is a trait strongly influenced by environmental factors (63), we cannot ignore the potential impact of parental origin (natural conditions) in relation to the hybrid offspring raised in the laboratory (laboratory conditions).

### More or less adapted conformation: How much did the wings change between parental species and hybrids?

The allometric effect was low for both sexes in the hybrids: in females, it was 15.7% and 5.6% for the first two factors, while in males, it was 12.5% and 9%, respectively. In addition, we compared the variance of shape, also referred to as MD, between the parental species and the hybrids, and no significant differences in metric disparity (MD = 0.00094) were observed. However, differences in wing morphology were detected, with hybrid males from ♀*Pc* × ♂*Ph* exhibiting the lowest variability (MD = 0.00058) (**Table 1**). Females of the ♀*Pc* × ♂*Ph* hybrid group exhibited thinner wings compared to their parental counterparts, potentially reflecting different flight strategies. Previous studies have suggested that varying wing morphotypes may influence flight behavior (37, 68). The observed shape differences, especially in hybrids, could have functional implications, possibly affecting flight capabilities and dispersal behavior. Similarly to what is observed in *Apis mellifera*, we can infer that differences in wing conformation in hybrids may impact and potentially alter their aerodynamics (69, 70).

**Table 1:** The metric disparity (MD) of wing shape in the parental species (*P. chinai* and *P. howardi*) and hybrid groups.

Species	Females (*n*)	Significance	Males (*n*)	Significance
*Panstrongylus chinai*	0.00074 (38)	ns	0.00094 (53)	ns
*Panstrongylus howardi*	0.00089 (36)	ns	0.00077 (47)	ns
♀*Pc* × ♂*Ph*	0.00085 (8)	ns	0.00058 (20)	ns
♀*Ph* × ♂*Pc*	0.00089 (26)	ns	0.00086 (34)	ns

**Figure 4:**
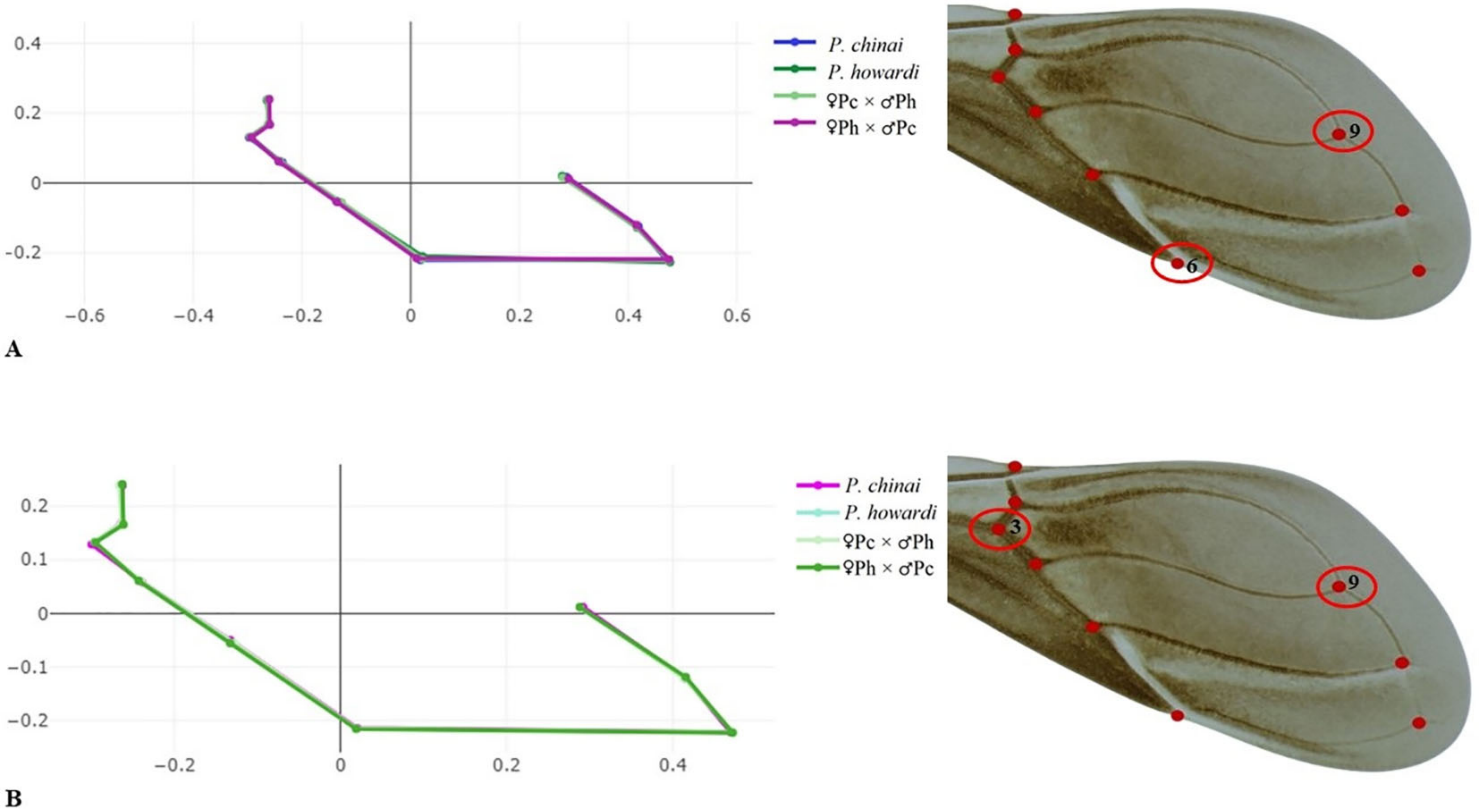
The figure presents the average wing shapes based on residual coordinates, illustrating the differences between females and males of the parental species (*P. chinai* and *P. howardi*) and the hybrid groups. **(A)** Females; **(B)** males.

The average objects (based on the residual coordinates) showed shape changes between *P. chinai* and *P. howardi* (parental), and the hybrids (♀*Pc* × ♂*Ph* and ♀*Ph* × ♂*Pc*), specifically related to the length of the membranous part of the wing and the width of the coriaceous part. In females, the primary difference was located at *landmark* 6 (the crossing between the radial and subcostal veins), while in males, it was at *landmark* 3 (the crossing between the middle radial and ulnar veins). Moreover, in females and males, *landmark* 9 (the crossing between the cubital and postcubital veins) exhibited notable differences, particularly between *P. chinai* and ♀*Ph* × ♂*Pc* in males, and *P. howardi* and ♀*Ph* × ♂*Pc* in females. In the latter case, this hybrid (♀*Ph* × ♂*Pc*) displayed a longer and narrower wing (**Figure 4**).

### Hybrids vs. parental species: is differentiation possible? Classification

For the classification: (i) The smallest Euclidean distance was observed between *P. howardi* and the ♀*Pc* × ♂*Ph* in females (0.01444), while in males, the smallest Euclidean distance was between the two hybrid groups (0.00954). This suggests that hybrid males may exhibit phenotypic characteristics that blur the boundaries between parental species and their hybrids (**Table 2**). This result highlights a trend where hybrids could potentially be considered a single phenotypic group, particularly when classified based on wing size and shape. (ii) The DA (validated reclassification) produced better results when classification was based on parental species rather than the hybrids, with scores generally higher for males (75%, 87%, 45%, 74%) than for females (74%, 64%, 38%, 58%). (iii) The *K*-means algorithm for wing shape successfully classified the parental species and hybrid groups, identifying an optimal *k*-value of 5 for females and 4 for males. While the *K*-means algorithm for wing size classified the groups with an optimal *k* = 3 in both females and males, conformation provided better classification, whereas size failed to distinguish between the two hybrid groups (*k* = 3, with parental species and a single hybrid group). This limitation may be due to the initial samples (parental) originating from different locations, leading to a higher-than-expected *k*. A similar situation was observed in Hernández et al. (37), where the lower *k*-value could be attributed to the uniform conditions in laboratory breeding, which tend to minimize potential differences.

**Table 2:** Discriminant analysis (Mahalanobis distances) and principal component analysis (Euclidean distances) were performed between the parental species (*P. chinai* and *P. howardi*) and hybrid groups.

	Females				Males			
	*P. chinai*	*P. howardi*	♀Pc × ♂Ph	♀Ph × ♂Pc	*P. chinai*	*P. howardi*	♀Pc × ♂Ph	♀Ph × ♂Pc
Principal component analysis (PCA): Euclidean distances								
*P. chinai*	0	0.02152	0.01757	0.01673	0	0.02248	0.01207	0.01153
*P. howardi*	0.02152	0	0.01444	0.02316	0.02248	0	0.02121	0.02165
♀*Pc* × ♂*Ph*	0.01757	0.01444	0	0.02045	0.02248	0.02121	0	0.00954
♀*Ph* × ♂*Pc*	0.01673	0.02316	0.02045	0	0.01153	0.02165	0.00954	0
Discriminant analysis (DA): Mahalanobis distances								
*P. chinai*	0	2.896^***^	2.257^**^	2.222^***^	0	3.376^***^	2.639^***^	2.547^***^
*P. howardi*		0	1.508	1.946^***^		0	3.049^***^	3.153^***^
♀*Pc* × ♂*Ph*			0	1.965^***^			0	1.687^***^
♀*Ph* × ♂*Pc*				0				0

^*^ *p* < 0.05; ^**^ *p* ≤ 0.01; ^***^ *p* ≤ 0.001.

When analyzing the morphological similarities of the groups using Euclidean and Mahalanobis distances, the highest similarity is observed between each parent and the hybrid in which that parent is the male in the cross. Specifically, *P. chinai* shows greater similarity with the hybrid ♀*Ph* × ♂*Pc*, while *P. howardi* is more similar to the hybrid ♀*Pc* × ♂*Ph* in both females and males. This raises the question: Does the male exhibit greater phenotypic dominance in wing conformation?

### General considerations

According to Vicente et al. (42), the hybrids present intermediate patterns, which could provide greater fitness than their parental species in: (i) the invasion process (domiciliary and peridomiciliary), (ii) shorter life cycles, (iii) feeding and defecation patterns (transmission dynamics of *T. cruzi*). These patterns may be similar to or indicate a higher susceptibility to *T. cruzi* infection than their parental species under laboratory conditions (71), highlighting the possible vector competence of these insects for Chagas disease. While *P. chinai* is distributed in the Loja and El Oro provinces (14, 23), *P. howardi* is restricted to Manabí province (21). Despite search efforts, no hybrid individuals have been found in nature between these regions, even after conducting predictive models through ecological niche modeling (14). In addition, for *P. chinai*, *P. howardi*, and their hybrids (♀*Pc* × ♂*Ph* and ♀*Ph* × ♂*Pc* crosses), no significant differences were observed in the preoviposition period or the average number of eggs (14). However, *P. chinai* could be considered a univoltine species, as its complete life cycle takes 371.4 days ± 22.3 days (20), whereas *P. howardi* completes its life cycle in 186.9 days ± 12.2 days (Castillo et al., unpublished data), nearly half the duration of *P. chinai*. In the transmission dynamics of *T. cruzi*, *P. chinai* presented a natural infection rate of 14% (23), while *P. howardi* had a rate of 53.2% (21). No information on the life cycle or infection index of the hybrids has been reported so far. Nevertheless, anthropization and climate change can impact the incidence, seasonal transmission, and geographic distribution of vectors and the diseases they transmit (72–74). Changes to the ecological landscape pose an epidemiological risk for the emergence of endemic diseases and may facilitate the convergence of different species, leading to the formation of natural hybrids.

Both size and shape contribute to understanding the similarities and differences between groups. While size is a useful characteristic for discrimination, shape offers greater resolution and is less influenced by environmental variation (75). *Panstrongylus chinai* displays a notably dark coloration, allowing it to hide in dark places such as under beds and holes within adobe walls inside domiciles. It is also commonly found in peridomiciliary habitats, including chicken coops, as well as pigeon and rat nests (23). On the other hand, *P. howardi* exhibits a distinct orangish coloration that enables it to camouflage effectively among bricks (21). However, hybrids (♀*Pc* × ♂*Ph* and ♀*Ph* × ♂*Pc* crosses) of both sexes generally displayed a coloration characteristic of the *P. howardi* phenotype. In addition, *P. chinai* exhibited the highest variability in CS and shape, which may be attributed to the geographical range where the specimens were collected (five communities in Loja province at altitudes ranging from 700 to 1,400 masl). Furthermore, abiotic and biotic conditions of breeding habitats can influence the wing geometry through carry-over effects from the immature to the adult stage. Individuals from a single sampling location would likely reduce shape variation within each species, potentially leading to an overestimation of actual interspecific variation.

This study found that the greatest Euclidean distance (indicating the most dissimilar phenotype) was between *P. howardi* and the ♀*Ph* × ♂*Pc* hybrid. This suggests that *P. chinai* exerts greater phenotypic influence when the male is the parental specimen. Despite this, DA based on parental species generally outperformed hybrid-based classification, with males showing better classification results than females. Overall, the hybrids’ size and shape tended to resemble *P. chinai*, though this was not always the case.

Considering these species as “secondary”—*P. chinai* in Loja and *P. howardi* in Manabí—we recommend further expanding knowledge of the genus *Panstrongylus* in Ecuador and Latin America. Geometric morphometrics has proven to be a highly useful and cost-effective technique for identifying sibling species and hybrids. Further research should explore additional morphological traits (i.e., head, pronotum, and antennal phenotype) and molecular markers, incorporating integrative approaches (7, 8, 14) to elucidate the evolutionary and ecological dynamics of triatomine hybridization (76, 77).

## Conclusion

In summary, our study confirms the utility of geometric morphometrics in distinguishing *P. chinai*, *P. howardi*, and their hybrid offspring. The hybrids tend to display traits more closely related to one of the parental species, but differences in wing size and shape suggest potential shifts in flight behavior and overall fitness. Hybridization may not always confer evolutionary benefits; therefore, its ecological implications in this genus warrant further investigation, particularly regarding its role in vector-borne disease transmission. Future research should explore additional morphological, behavioral, and molecular traits to fully understand the dynamics of hybridization and its impact on the fitness and spread of these species.

## Data Availability

The original contributions presented in the study are included in the article/supplementary material. Further inquiries can be directed to the corresponding authors.
